# The Effect of Acute Sleep Extension on Blood Pressure Is Dependent on the Change in Sleep Efficiency

**DOI:** 10.3390/clockssleep6040036

**Published:** 2024-10-04

**Authors:** Joaquin U. Gonzales, Cayla Clark, Jacob R. Dellinger

**Affiliations:** Department of Kinesiology & Sport Management, Texas Tech University, Lubbock, TX 79409, USA; cclark33@twu.edu (C.C.); jacob.dellinger@ttu.edu (J.R.D.)

**Keywords:** sleep duration, sleep extension, blood pressure, microvascular function, reactive hyperemia, aging

## Abstract

The present study investigated the effect of acute sleep extension on blood pressure and microvascular vasodilation. Sleep and daily physical activity were objectively measured at home for two weeks using wrist actigraphy in 22 adults (60 ± 15 y). Vascular measurements were made in the morning on the 8th and 15th day. Participants spent at least 10 h in bed on the night prior to one of these testing days to extend sleep. Mean arterial blood pressure (MAP) and peak reactive hyperemia in the forearm were measured on each testing day. Reactive hyperemia and MAP were unaltered (*p* > 0.05) by sleep extension in the total sample. However, adults who experienced improved sleep efficiency with sleep extension (n = 10, 4.2 ± 1.4%) exhibited reduced MAP (−5.5 ± 4.6 mm Hg, *p* = 0.005) while adults who had little change or decreased sleep efficiency (n = 12, −1.7 ± 2.9%) showed no change in MAP. The reduction in MAP was significantly different between sleep efficiency groups (*p* = 0.005, Hedges’ *g* = 1.21) after adjustment for sex and moderate-to-vigorous physical activity. The results of this study suggest that sleep extension has the potential to reduce blood pressure in midlife to older adults when the additional sleep time improves the quality of sleep.

## 1. Introduction

The U.S. Center for Disease Control recommends 7 h or more of sleep per night to achieve “optimal health and wellbeing” [1]. This recommendation stems partly from data showing that the lowest risk of total cardiovascular disease is associated with a sleep duration of 7 h per night [2]. Unfortunately, around 30% of the U.S. population sleep less than 7 h per night, with this proportion of adults having a greater prevalence of hypertension than adults who meet the recommended amount of sleep [3]. Middle-aged to older adults are of particular interest as they have lower sleep durations than younger adults [4] and suffer from higher rates of cardiovascular disease [5]. Therefore, more research is needed in this population to understand the impact of sleep on vascular health.

Research investigating acute perturbations in sleep provides insight into the early effects of altered sleep duration on biological function. For instance, one night of sleep restriction or full sleep deprivation is found to impair endothelial-dependent vasodilation of the microvasculature [6], blunt microvascular reactivity [7], and elevate blood pressure [8,9]. Few studies, however, have examined the effect of acute sleep extension on biomarkers of vascular health. One study showed no effect of longer sleep durations over the weekend on blood pressure measured the following Monday [10]. However, this study was limited by a sample of young adults with habitually short sleep durations and the researchers did not account for physical activity which can influence next-day blood pressure [11]. Other studies have shown one to three nights of extended sleep lowers inflammation and improves immune function when it follows a period of sleep restriction [12,13]. Thus, data exist to support a potential positive influence of acute sleep extension on modulating factors important for vascular health, particularly in adults with a sleep debt, but further investigation is still needed to understand the effect of sleep extension on vascular function under normal conditions.

The aim of this study was to determine the impact of acute sleep extension on blood pressure and microvascular function in middle-aged to older adults. Our rationale for focusing on adults within these age categories is that they typically sleep less than the recommended 7 h per night and have low sleep efficiency [4]. Thus, combined with age-related vascular dysfunction [14], we postulated that this population would be ideal to observe a positive effect of acute sleep extension on vascular function if one was to be present. We hypothesized that sleep extension would improve vascular health as evidenced by lower blood pressure and greater microvascular vasodilation, but that the improvement would be dependent upon whether sleep extension improved the quality of sleep (i.e., sleep efficiency). While sleep extension increases sleep duration, it also has the potential to increase wake after sleep onset as more time is spent in bed [15,16]. More time spent awake at night lowers sleep efficiency [17,18], which has the potential to influence blood pressure as past studies find an inverse relationship between sleep efficiency and blood pressure in middle-aged adults [19,20]. Therefore, our hypothesis was that vascular function would be improved in adults who experienced improved sleep efficiency with sleep extension.

## 2. Results

### 2.1. Demographics

In the total sample (N = 22), on average, participants were 60 ± 15 y, had a body mass index of 25 ± 6 kg/m^2^, and a seated brachial systolic and diastolic blood pressure of 123 ± 16 and 75 ± 8 mm Hg, respectively. There were six women and four men (n = 10) who exhibited improved (≥2%, range 2.3 to 6.9%) sleep efficiency after one night of extended sleep, and ten women plus two men (n = 12) who showed little-to-no change or decreased sleep efficiency (<2%, range −8.2 to 1.3%). There was no difference in age (improved vs. no change: 60 ± 16 vs. 61 ± 16 y, *p* = 0.95), body mass index (27 ± 8 vs. 24 ± 4 kg/m^2^, *p* = 0.48), seated systolic blood pressure (123 ± 16 vs. 122 ± 16 mm Hg, *p* = 0.86), and seated diastolic blood pressure (76 ± 7 vs. 74 ± 9 mm Hg, *p* = 0.74) between sleep efficiency groups.

### 2.2. Effect of Acute Sleep Extension on Sleep and Physical Activity Parameters

As shown in Table 1 for the entire sample, acute sleep extension increased time in bed (*p* < 0.001), sleep duration (*p* < 0.001), and number of awakenings (*p* ≤ 0.05) as compared to their 13-night average or the day prior to vascular testing under the condition of usual sleep. The added time in bed on the night of sleep extension resulted in less time spent in light physical activity as compared to their 13-day average (*p* = 0.01) or the day prior to vascular testing under the condition of usual sleep (*p* = 0.02). There was no effect (*p* > 0.05) of sleep extension on the average time per awakening or amount of time awake after sleep onset. Moderate-to-vigorous physical activity and daily step count were also similar (*p* > 0.05) on the day of sleep extension compared to the 13-day average. Sleep efficiency was slightly higher on the night of acute sleep extension compared to the night of usual sleep prior to vascular testing (*p* = 0.04), but sleep efficiency was similar on the night of sleep extension compared to the 13-night average.

Table 2 shows the effect of acute sleep extension on sleep and physical activity in adults divided into groups based on the change in efficiency. The 13-night average was used for comparison purposes as it reflects their habitual sleep and did not differ from the usual night prior to testing (Table 1). Time in bed and sleep duration were similar between groups in terms of their 13-night averages as well as the significant increase in time in bed (main effect for time: 164 ± 45 min, *p* < 0.001) and sleep duration (main effect for time: 144 ± 42 min, *p* < 0.001) with acute sleep extension. Both groups showed an increase in the number of awakenings per night after sleep extension (main effect for time: 4.4 ± 5.6, *p* = 0.002). However, the group with improved sleep efficiency spent less time awake per awakening during sleep extension (−0.56 ± 0.52 min, *p* = 0.02), while the group with no improvement in sleep efficiency spent more time awake per awakening (0.72 ± 0.96 min, *p* = 0.005). This resulted in an increase in wake after sleep onset for the group who exhibited no improvement in sleep efficiency that was significantly greater (mean difference of 34 ± 26 min, *p* = 0.01) than the group with improved sleep efficiency.

### 2.3. Effect of Acute Sleep Extension on Vascular Function

In the total sample, acute sleep extension had no effect (all *p* > 0.05) on blood pressure or microvascular function (Table 3). In contrast, group analysis showed that systolic blood pressure (brachial systolic blood pressure: −6.7 ± 7.3 mm Hg, *p* = 0.01; central aortic systolic blood pressure: −6.7 ± 6.3 mm Hg, *p* = 0.007), diastolic blood pressure (−3.4 ± 3.3 mm Hg, *p* = 0.01), and mean arterial pressure (MAP) (−5.5 ± 4.6 mm Hg, *p* = 0.005) were reduced after sleep extension in adults who exhibited improved sleep efficiency (Table 4). A similar effect was not observed in the group of adults who showed little change or decreased sleep efficiency with acute sleep extension. The change in systolic blood pressure (pooled sample, brachial systolic blood pressure: r = −0.44, *p* = 0.03; central aortic systolic blood pressure: r = −0.45, *p* = 0.03) and MAP (r = −0.47, *p* = 0.02) was negatively correlated with the change in sleep efficiency. Meanwhile, the change in diastolic blood pressure was positively correlated with the change in nocturnal awakenings with sleep extension (r = 0.45, *p* = 0.03).

**Figure 1:**
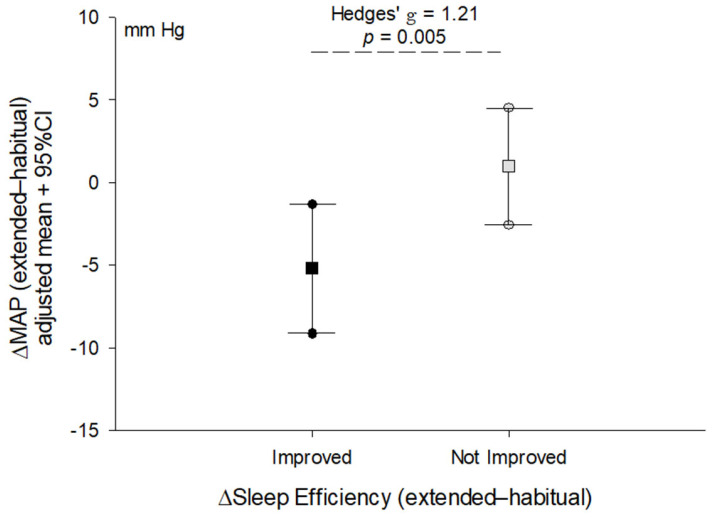
Comparison of the change (Δ) in mean arterial pressure after acute sleep extension between adults who improved sleep efficiency (≥2% change) and those who exhibited little change or a decrease in sleep efficiency (<2% change). Line plot shows the mean (square) and 95% confidence intervals (circles) for the change in blood pressure adjusted for sex and time spent in moderate-to-vigorous physical activity on the day of extended sleep. Effect size reported as Hedges’ *g* due to different sample sizes between groups. *p*-value reflects the difference between groups derived from ANCOVA.

After adjusting for sex and time spent in moderate-to-vigorous physical activity on the day of extended sleep, the change in brachial systolic blood pressure (*p* = 0.13) and central aortic systolic blood pressure (*p* = 0.059) was no longer statistically significant. In contrast, the change in diastolic blood pressure (adj. mean [95%CI], −3.8 [−6.4, −1.3] vs. 2.1 [−0.1, 4.4], *p* = 0.002, Hedges’ *g* = 1.56) and MAP (Figure 1) after sleep extension remained significantly different between groups after adjusting for sex and time spent in moderate-to-vigorous physical activity on the day of extended sleep.

## 3. Discussion

The objective of this study was to determine the effect of acute sleep extension on peripheral vascular function in middle-aged to older adults. These age groups are reported to sleep less than younger adults and suffer from age-related attenuation in sleep efficiency [4]. The negative effect of short sleep durations on vascular function is well documented [21]; thus, we hypothesized that one night of extended sleep in this population would yield a positive effect on established markers of vascular function. This study found that in the total sample of adults acute sleep extension did not impact blood pressure or microvascular vasodilation despite increasing sleep duration by around 2.5 h on average. It was only in adults who had an improved sleep efficiency with sleep extension that we observed a positive effect on blood pressure. This acute effect of sleep extension on reducing blood pressure supports the notion that sleep is important for cardiovascular health.

One other investigation has examined the impact of acute sleep extension on blood pressure in adults. Kubo and colleagues [10] reported no effect of sleep extension performed over the weekend on brachial systolic and diastolic blood pressure measured the following Monday in a sample of younger adults with habitually short sleep durations. The similarities of the present study with this previous report are that sleep duration was extended by around 2 h per night, on average, and sleep efficiency was not changed by sleep extension in the entire sample. Moreover, the present study and the work by Kubo and colleagues [10] found no impact of acute sleep extension on blood pressure when the total sample was analyzed. However, the present study demonstrates that the effect of sleep extension on blood pressure may be dependent on how the extra time in bed trying to sleep alters sleep efficiency. Sleep efficiency reflects the percent time spent asleep relative to time in bed with higher values considered greater sleep quality. Previous studies have reported that sleep efficiency is inversely associated with morning resting blood pressure in middle-aged adults [19,20]. Our results are consistent with this cross-sectional data, but we show in an experimental study design that improved sleep efficiency during acute sleep extension can lower blood pressure. The change in sleep efficiency with sleep extension was negatively correlated with the change in systolic blood pressure and MAP, further supporting a link between sleep efficiency and resting blood pressure.

The total sleep time reached during the night of extended sleep was 9 h on average for the total sample and in the two sleep efficiency groups. This total sleep time is considered a ‘long sleep duration’ by the American Heart Association [22] and is associated with negative cardiovascular outcomes including hypertension in adults who report the regular habit of sleeping for long durations [3,23]. The present study did not observe a negative effect of long sleep durations on vascular function. This is not surprising considering the acute nature of this study. However, an interesting observation was that the positive effect of sleep extension on blood pressure occurred despite long sleep durations being reached by our participants. These data provide evidence that a single night of long sleep duration, in itself, is not detrimental to peripheral vascular health. Rather, our data support the position that a long sleep duration has the potential to negatively impact vascular health through its influence on sleep fragmentation (i.e., awakenings and wake after sleep onset) [24]. The extra time in bed required to sleep longer durations in the present study led to a greater number of awakenings, as shown in Table 1 and Table 2. Notably, the change in number of awakenings was positively correlated with the change in diastolic pressure. In adults who experienced little change or decreased sleep efficiency, the number of awakenings increased as well as the time spent awake per awakening. This resulted in around 30 added minutes of wake after sleep onset relative to their usual amount. In contrast, the group that experienced improved sleep efficiency (and lower blood pressure) showed no change in wake after sleep onset with sleep extension. This information illustrates the importance of sleep fragmentation as a factor that may partly determine how acute sleep extension, even when long sleep durations are reached, influences vascular health.

The mechanism by which acute sleep extension reduced blood pressure in adults who showed improved sleep efficiency is not clear from our data. The lack of change in microvascular vasodilation (peak forearm blood flow and vascular conductance) indicates arterial vasodilatory capacity was not improved by the one night of extended sleep. This runs counter to our recent study that found that five consecutive nights of sleep extension increased microvascular vasodilation in middle-aged adults [16]. This discrepancy may be due to different intervention periods (one vs. five nights), age groups examined, and/or control conditions. Sleep extension was compared to each participant’s usual or habitual sleep in the present study while in our past investigation we limited the time in bed to 8 h for a comparison to a control condition. Another variable unchanged by acute sleep extension in the present study was augmentation pressure. This indicates that pressure wave reflection and its ability to alter central aortic pressure beyond the first systolic shoulder was not a major factor that contributed to lower systolic blood pressure after extended sleep. Heart rate was also unchanged, suggesting cardiac autonomic function was likely not influential. Although we cannot fully discount this possibility since sleep extension has been reported to be associated with lower cardiac sympathetic activity and/or elevated parasympathetic activity in the morning hours as compared to short sleep [25]. Considering the significant reduction in diastolic and MAP in adults with improved sleep efficiency, we must postulate that total peripheral resistance was lower after extended sleep. Mean arterial pressure is a primary determinant of total peripheral resistance alongside cardiac output. Our data suggest that cardiac output was unaffected by sleep extension as heart rate and forearm blood flow was unchanged, but this postulation requires confirmation in future research that includes direct measures of central hemodynamics and sympathetic nerve activity.

An important component of this study is the objective measurement of daily physical activity using waist-worn accelerometers. We did not find daily physical activity to differ on the days prior to testing, that is, before the usual night or extended night of sleep (Table 1). This is important as physical activity performed during the day prior to sleep can alter sleep quality in middle-aged and older adults [26]. Additionally, moderate-intensity exercise can lower blood pressure measured the morning after exercise [11]. Thus, it is important to account for physical activity when investigating the effect of altered sleep on vascular function. In the present study, adults in the group with improved sleep efficiency with acute sleep extension tended to spend more time in moderate-to-vigorous physical activity than adults who showed little change or decreased sleep efficiency (Table 2). Therefore, adjusting for physical activity in our comparisons of blood pressure between groups was needed. We observed that time spent in moderate-to-vigorous physical activity on the day of extended sleep partly explained the variance in the change in systolic blood pressure following extended sleep. Meanwhile, the reduction in diastolic blood pressure and MAP was independent of physical activity.

### 3.1. Limitations

This study included a small sample size of relatively healthy adults without a history of stroke, myocardial infarction, or diabetes mellitus. This reduces the generalizability of the present findings due to the high prevalence of these conditions in the general population. Nevertheless, the middle-aged to older adults sampled in this study had an average sleep duration (~6.5 h) aligned with what is reported in late adulthood [4], and therefore, the results are relevant to this population. Another limitation is the acute nature of this study. Our aim was to investigate how the body responds to an extended night of sleep. It is from a scientific perspective that we conducted this study. However, we acknowledge that one night of extended sleep may not reflect changes in biological function that may occur with longer term (weeks to months) sleep extension. Indeed, the literature in middle-aged to older adults regarding blood pressure changes following six weeks of sleep extension are mixed [27,28], and likely depend on population characteristics (e.g., mental health status, underlying disease, medications, etc.) [24]. Lastly, our data do not supply information about what made one group of participants experience improved sleep efficiency during extended sleep while others did not. While usual sleep patterns were similar between groups, we did not assess subjective sleepiness or sleep architecture (e.g., REM, non-REM, and slow-wave activity), which may have helped explain different responses to acute sleep extension. For instance, healthy young adults with excessive daytime sleepiness show a decrease in sleep efficiency with one night of sleep extension [18]. Additionally, past research reports increased REM and non-REM sleep with a single night of sleep extension [29], but extended sleep contains a low amount of slow-wave activity [30]. This may vary between individuals, thus impacting wakefulness throughout the night.

### 3.2. Conclusions

In summary, this study used wrist actigraphy to monitor sleep in middle-aged to older adults to determine the acute effect of sleep extension on vascular function. We observed morning blood pressure to be significantly reduced after one night of sleep extension in adults who had improved sleep efficiency. This result highlights the impact of sleep efficiency on the potential for sleep extension to improve vascular function when extending sleep. This acute effect of sleep on reducing blood pressure should be replicated in future research that includes direct measures of central hemodynamics and cardiac autonomic function across a longer assessment period to understand the temporal pattern of change in blood pressure following extended sleep.

## 4. Materials and Methods

### 4.1. Participants

Twenty-two (16 women, 6 men) adults between the ages of 40 and 86 years participated in this study. Twelve of the sixteen women reported being post-menopausal. Two post-menopausal women reported being on hormone (estrogen) replacement therapy. Four of the sixteen women reported being pre-menopausal with normal menstrual cycles and were not taking oral contraceptives. Menstrual cycle was not accounted for in this study as resting blood pressure [31] and microvascular function [32] are not found to be significantly influenced by menstrual cycle phase in pre-menopausal women. Of the six men, three reported being on statin therapy (Atorvastatin) and one reported taking antihypertensive medication (Lisinopril). Participants were included if they were 40 years or older and reported no previous physician-diagnosed sleep-related disorders (e.g., sleep apnea). Exclusion criteria included taking sleep medication or related supplements, night shift work, or evidence of insomnia using the Insomnia Symptom Questionnaire (ISQ). Participants were also excluded if they reported being a smoker, a vaper, or having a personal history of stroke, myocardial infarction, or diabetes mellitus.

### 4.2. Study Design

This experimental study employed a crossover design that asked participants to keep their usual sleep schedule for two weeks. Sleep was monitored at home using wrist actigraphy and physical activity was monitored by a waist-worn accelerometer. One night during the two-week period, participants were instructed to spend at least 10 h in bed trying to sleep (i.e., sleep extension). This night was randomized between participants and was either the night of the 7th or 14th day during the two-week period. The order was counter-balanced and randomized using Excel’s randomize function. Excluding the one night of extended sleep, the other 13 nights were averaged together to reflect participants’ usual sleep behavior. Vascular testing occurred in the morning after the 7th and 14th night. Participants arrived for testing after an overnight fasting period of at least 8 h that included no caffeine. All study visits were scheduled at the same time in the morning for each participant to reduce the effect of diurnal fluctuation in vascular function.

### 4.3. Sleep Monitoring

A triaxial accelerometer (GT9X, ActiGraph, Pensacola, FL, USA) sampling at 30 Hz was worn by participants on their non-dominant wrist throughout the study and they were advised only to remove accelerometers when bathing or swimming. Accelerometer data were reduced to 60 s epochs before being analyzed using a combination of Cole–Kripke and Tudor-Locke algorithms in ActiLife sleep analysis software (version 6.13.3, ActiGraph, Pensacola, FL, USA). Time in bed, sleep duration, sleep efficiency [(duration/time in bed) × 100], and wake after sleep onset were recorded from the analysis. This accelerometer technology (Actigraph), sensor location (wrist), and these data processing techniques have been shown by others to compare well with polysomnography measures of sleep duration, wake after sleep onset, and sleep efficiency [33]. On nights that the ActiLife sleep analysis identified questionable sleep periods, researchers referred to a sleep diary kept by participants to help identify bedtimes and waketimes in order to reduce actigraphy overestimation of sleep time [34].

### 4.4. Physical Activity Monitoring

Daily moderate-to-vigorous physical activity was measured using a waist-worn triaxial accelerometer (GT3X, ActiGraph, Pensacola, FL, USA) positioned over the left hip with an adjustable elastic belt. Data were sampled at 30 Hz and then reduced to 60 s epochs for analysis after undergoing wear-time validation using the Choi equation to ensure wear periods less than 600 min were excluded from the analysis. Vector magnitude data were analyzed for minutes per day spent in light and moderate-to-vigorous physical activity using the Freedson Adult VM3 cut points of 0–2689 counts per minute for light activity and 2690 counts per minute as the transition point to at least moderate-intensity physical activity [35]. Total steps taken per day were also recorded.

### 4.5. Blood Pressure and Heart Rate

Blood pressure waveforms were measured at the right radial artery using applanation tonometry (SphygmoCor PVx, AtCor Medical, Sydney, Australia) after participants rested in the supine position for 10 min. An ensemble-averaged radial artery pressure waveform was calibrated to the average of at least three brachial blood pressure measurements taken using an automated device (HEM-907XL, Omron Healthcare, Kyoto, Japan) clinically validated in adults [36]. Diastolic and mean blood pressure were used for calibrating the pressure waveform as these pressures are relatively constant across the arterial tree. A general transfer function was used to synthesize a central aortic waveform from the radial artery pressure waveform that allowed for estimation of central aortic systolic, augmentation pressure, and end-systolic blood pressure. Augmentation pressure is the increment in central aortic blood pressure above its first systolic shoulder and reflects pressure wave reflection. End-systolic pressure was used in the present study as a surrogate for mean arterial pressure (MAP) as it reflects left ventricular pressure at the end of systole. Heart rate was derived from the frequency of radial pressure waves during data collection.

### 4.6. Microvascular Vasodilation

Venous occlusion plethysmography (EC6 system, Hokanson, Bellevue, WA, USA) was used to measure peak reactive hyperemia in the forearm after at least 15 min of supine rest. The left forearm was elevated approximately 10 cm above heart level and fitted with a strain gauge placed around the widest portion of the forearm. A small blood pressure cuff was wrapped around the left wrist, and a medium-sized blood pressure cuff was placed around the upper arm. The medium-sized cuff was controlled by a rapid inflator (model E20, Hokanson, Bellevue, WA, USA). During the forearm blood flow (FBF) measurement, the wrist cuff was inflated to 200 mmHg to occlude hand circulation while the arm cuff inflated intermittently to 50 mmHg to stop venous outflow from the arm. With these settings, four FBF measurements could be obtained within one minute. Peak FBF was measured during a three-minute period that followed 10 min of ischemia induced by inflating the arm cuff to 220 mmHg. Beat-by-beat blood pressure was measured throughout the protocol (CNAP monitor 500at, CNSystems, Graz, Austria) so peak forearm vascular conductance (FVC, FBF/mean blood pressure) could be calculated.

### 4.7. Statistical Analysis

Two-tailed paired t-tests were used to test for differences in vascular variables when assessing data from all participants. A one-way repeated measures analysis of variance (ANOVA) was used to compare sleep and daily physical activity variables across three time points: usual sleep (across 13 nights), the usual night of sleep prior to testing, and the extended night of sleep prior to testing. A Friedman repeated measures ANOVA on ranks was used when data failed normality using a Shapiro–Wilk test. Groups were created based on the change in sleep efficiency following extended sleep relative to the 13-night average. The median change was 1.3%; thus, a ≥2% cutoff was used to divide the adults into groups. Two-tailed independent-sample t-tests were used to examine differences in demographics between groups. A two-way repeated-measures ANOVA was used to compare vascular variables between sleep efficiency groups (≥2 vs. <2% efficiency) and time (usual vs. extended sleep). Analysis of covariance (ANCOVA) was used to compare the change in blood pressure between groups when controlling for confounding variables. Bonferroni post hoc testing was used for all ANOVA and ANCOVA tests upon significant main effects or interactions. Hedges’ *g* effect sizes were calculated when the change in vascular function differed between sleep efficiency groups. Effect sizes of 0.2, 0.5, and 0.8 are considered small, medium, and large, respectively. Lastly, Pearson correlations were performed to assess relationships between the change in sleep and vascular function following extended sleep. Statistical significance was set a priori at *p* ≤ 0.05. Statistics was performed using SigmaPlot (version 13, Systat Software, San Jose, CA, USA). Data are presented as mean ± SD unless otherwise stated.

## Figures and Tables

**Table 1:** Sleep parameters and daily physical activity in total sample (N = 22).

	13-Night Average	Usual Night Prior to Testing	Sleep Extension	*p*-Value
***Sleep parameters***				
Time in bed (min)	468 ± 52	452 ± 71	633 ± 41 ^a,b^	<0.001
Sleep duration (min)	405 ± 45	386 ± 61	549 ± 36 ^a,b^	<0.001
Efficiency (%)	86 [84, 89]	85 [81, 90]	88 [82, 91] ^b^	0.04
WASO (min)	56 [45, 71]	59 [37, 87]	67 [49, 107]	0.10
Awakenings (#)	22 [16, 25]	18 [14, 25]	27 [17, 31] ^a,b^	0.009
Time per awakening (min)	2.7 [2.3, 3.4]	3.2 [2.4, 3.9]	2.7 [2.1, 3.8]	0.66
***Physical activity parameters***				
Light (min per day)	868 [801, 916]	873 [755, 952]	760 [658, 841] ^a,b^	0.007
MVPA (min per day)	23 [13, 40]	18 [7, 42]	17 [6, 49]	0.61
Total steps (per day)	6437 ± 2920	6198 ± 3249	6304 ± 3925	0.91

Values are mean ± SD or median [interquartile range]. WASO, wake after sleep onset. MVPA, moderate-to-vigorous physical activity. ^a^, difference with 13 days of usual sleep (*p* < 0.05); ^b^, difference with usual night of sleep prior to testing (*p* < 0.05).

**Table 2:** Sleep and daily physical activity in adults divided based on the change in sleep efficiency after acute sleep extension.

	Improved (n = 10, 6 F 4 M)		Not Improved (n = 12, 10 F 2 M)		2-Way RM ANOVA		
	13-Night Average	Sleep Extension	13-Night Average	Sleep Extension	Group	Time	Int.
***Sleep parameters***							
Time in bed (min)	468 ± 50	625 ± 26	467 ± 55	638 ± 51	0.74	<0.01	0.47
Sleep duration (min)	405 ± 49	559 ± 34	405 ± 43	541 ± 38	0.56	<0.01	0.37
Efficiency (%)	86 ± 4	90 ± 3 *^,†^	86 ± 3	85 ± 5 *	0.12	0.02	<0.01
WASO (min)	60 ± 20	59 ± 28	60 ± 20	93 ± 43 *^,†^	0.18	<0.01	<0.001
Awakenings (#)	21 ± 4	24 ± 7	20 ± 6	26 ± 10	0.93	<0.01	0.16
Time per awakening (min)	2.7 ± 0.4	2.2 ± 0.4 *^,†^	3.0 ± 0.9	3.8 ± 1.5 *	0.02	0.63	<0.01
***Physical activity parameters***							
Light (min per day)	901 ± 152	784 ± 137	852 ± 53	739 ± 118	0.23	<0.01	0.94
MVPA (min per day)	38 ± 28	40 ± 31	24 ± 21	21 ± 23	0.12	0.85	0.56
Total steps (per day)	6636 ± 3129	7518 ± 4084	6271 ± 2864	5293 ± 3649	0.34	0.94	0.17

Values are mean ± SD. WASO, wake after sleep onset. MVPA, moderate-to-vigorous physical activity. *, different from 13-night average within group (*p* < 0.05); ^†^, different between groups (*p* < 0.05).

**Table 3:** Effect of acute sleep extension on vascular function in total sample (N = 22).

	Usual Sleep	Sleep Extension	*p* Value
Brachial systolic blood pressure (mm Hg)	126 ± 17	123 ± 16	0.13
Central aortic systolic blood pressure (mm Hg)	111 ± 15	109 ± 14	0.12
Diastolic blood pressure (mm Hg)	73 ± 7	72 ± 7	0.54
Mean arterial pressure (mm Hg)	101 ± 13	99 ± 12	0.18
Augmentation pressure (mm Hg)	12 ± 6	11 ± 7	0.07
Peak FBF (mL/100 mL/min)	24 ± 8	23 ± 8	0.22
Peak FVC (mL/100 mL/min/mmHg)	0.26 ± 0.10	0.27 ± 0.10	0.33

Values are mean ± SD. FBF, forearm blood flow. FVC, forearm vascular conductance.

**Table 4:** Effect of acute sleep extension on vascular function in adults based on the change in sleep efficiency.

	Improved (n = 10, 6 F 4 M)		Not Improved (n = 12, 10 F 2 M)		2-Way RM ANOVA		
	Usual Sleep	Sleep Extension	Usual Sleep	Sleep Extension	Group	Time	Int.
Heart rate (bpm)	64 ± 11	66 ± 12	62 ± 6	62 ± 5	0.64	0.52	0.53
Brachial SBP (mm Hg)	126 ± 16	119 ± 13 *	125 ± 18	126 ± 18	0.69	0.07	0.04
Central SBP (mm Hg)	112 ± 15	106 ± 12 *	111 ± 15	112 ± 16	0.71	0.058	0.02
Diastolic BP (mm Hg)	74 ± 5	71 ± 5 *	72 ± 8	73 ± 7	0.98	0.31	<0.01
MAP (mm Hg)	101 ± 12	96 ± 10 *	100 ± 14	102 ± 14	0.65	0.08	0.01
AP (mm Hg)	11 ± 6	9 ± 6	13 ± 7	13 ± 7	0.43	0.06	0.26
Resting FBF (mL/100 mL/min)	1.7 ± 0.8	1.5 ± 0.3	1.7 ± 0.8	1.4 ± 0.6	0.88	0.16	0.40
Peak FBF (mL/100 mL/min)	27 ± 8	27 ± 9	21 ± 8	22 ± 7	0.12	0.63	0.38
Peak FVC (mL/100 mL/min/mmHg)	0.29 ± 0.10	0.30 ± 0.10	0.23 ± 0.09	0.24 ± 0.09	0.15	0.31	0.53

Values are mean ± SD. SBP, systolic blood pressure. BP, blood pressure. MAP, mean arterial pressure. AP, augmentation pressure. FBF, forearm blood flow. FVC, forearm vascular conductance. *, different from usual night of sleep within group (*p* < 0.05).

## Data Availability

The data for the study presented in this article will be shared on reasonable request to the corresponding author.
